# Factors Predictive of Development of Acute Kidney Injury in Patients With COVID-19 in Brunei Darussalam

**DOI:** 10.7759/cureus.37230

**Published:** 2023-04-06

**Authors:** Aung Phyo Oo, Mohammad Nafiz Riaduzzaman, Mohamed Ibrahim Alsaman, Abdur Rahman Rubel, Jayakrishnan Pisharam, Muhammad Abdul Mabood Khalil, Chiao Yuen Lim, Vui Heng Chong, Jackson Tan

**Affiliations:** 1 Department of Nephrology, Raja Isteri Pengiran Anak Saleha (RIPAS) Hospital, Bandar Seri Begawan, BRN; 2 Department of Medicine, Raja Isteri Pengiran Anak Saleha (RIPAS) Hospital, Bandar Seri Begawan, BRN; 3 Department of Medicine, Pengiran Muda Mahkota Pengiran Muda Haji Al-Muhtadee Billah (PMMPMHAMB) Hospital, Tutong, BRN; 4 Pengiran Anak Puteri Rashidah Sa'adatul Bolkiah (PAPRSB) Institute of Health Sciences, Universiti Brunei Darussalam, Bandar Seri Begawan, BRN

**Keywords:** covid-19 infection, corona virus disease 2019, risk factors, sars-cov-2 infection, acute kidney failure

## Abstract

Introduction: Acute kidney injury (AKI) in coronavirus disease 2019 (COVID-19) patients affects their health outcomes. Incidence and outcomes varied in the literature, particularly with different population and epidemiological demographics. Data remain scarce in the Southeast Asia region. We report the incidence, outcomes, pattern, types of AKI, and factors that influence AKI patient outcomes in Brunei Darussalam.

Methods: All patients (N = 930) with COVID-19 who were admitted to the National Isolation Center (between 7th August 2021 and 30thSeptember 2021) were included in the study. The confirmation of AKI was based on the KDIGO (Kidney Disease Improving Global Outcomes) criteria.

Results: The mean age of the patients was 41.9 ± 14.4 years with diabetes mellitus (DM), hypertension (HT), and chronic kidney disease (CKD) accounting for 11.7%, 29.1%, and 4.8% of comorbidities, respectively. Overall, 109 (11.7%) had AKI (KDIGO Stage 1 [67.9%], 2 [13.8%], and 3 [18.3%]), while 75.2% of the cases occurred pre-admission and 26.6% were cases of acute exacerbation of CKD. Univariate analysis identified age (odd ratio [OR] 1.06), male gender (OR 1.63), local nationality (OR 8.03), DM (OR 4.44), HT (OR 5.29), vascular disease (OR 6.08), presence of gastrointestinal symptoms (OR 2.08), antibiotic (OR 3.70) and nephrotoxins exposures (OR 8.57) as significant variables. Multivariate analysis showed age (adjusted OR [AOR] 1.04), male gender (AOR 1.67), gastrointestinal symptoms (AOR 1.61), antibiotic (AOR 2.34), and nephrotoxins exposure (AOR 4.73) as significant.

Conclusions: Our study showed that one in nine patients with COVID-19 developed AKI with almost a third having stages 2 and 3 AKI. Older age, male gender, presence of GI symptoms, and antibiotic and nephrotoxin exposures were significant predictors of AKI. Patients with these factors should be prioritized for admission and treatment. Even though manifestations are generally now less severe, findings from this study can guide the management of COVID-19 as the disease enters the endemic stage. Furthermore, lessons learned from the COVID-19 pandemic will provide useful information and knowledge for future viral outbreaks or pandemics.

## Introduction

Coronavirus disease 2019 (COVID-19), caused by the severe acute respiratory syndrome coronavirus-2 (SARS-CoV-2), is predominantly a respiratory illness but can affect other organs. Kidney injury is a known complication of COVID-19, and the mechanism is directly related to the virus or, more commonly, secondary to hemodynamic and infective inflammatory consequences on the kidneys. Direct invasion of SARS-CoV-2 into kidney tissue causes acute tubular injury, and glomerular and vascular changes [1], or is related to the cytokine storm syndrome triggering tissue inflammation and local immune cell infiltration causing endothelial injury and microvascular thrombi [2]. Hypercoagulability can also foster the transition of acute tubular necrosis to cortical necrosis, rendering irreversible kidney failure [3]. Acute kidney injury (AKI) can lead to unfavorable outcomes, especially in patients with other risk factors.

AKI for hospitalized COVID-19 patients ranges between 0.5% and 36.6% in different studies [4,5], higher among patients requiring high dependency care [6,7]. Dialysis requirements varied between 14% and 22% [7,8]. A meta-analysis showed a pooled incidence of AKI of 19.5% (95% confidence interval [CI] 14.6-24.8%), while the pooled incidence of patients with AKI requiring kidney replacement therapy (KRT) was 39.0% (95% CI 16.4-64.6%) [9]. The mortality of patients with AKI was 54.2% (95% CI 44.7-63.6%) [10]. Another meta-analysis of 54 studies showed a higher pooled prevalence of AKI at 28% (95% CI 22-34%), but a much lower pooled prevalence of KRT of 9% (95% CI 7-11%) [10]. The largest prospective study to date, from the International Severe Acute Respiratory Infection Consortium (ISARIC), showed a slightly higher prevalence of AKI at 31.5% (n = 13,000/41,2954) [11]. These meta-analyses showed that male gender, older age, smoking, obesity, hypertension (HT), diabetes mellitus (DM), pneumonia, cardiovascular disease, cancer, chronic kidney disease (CKD), mechanical ventilation, and use of vasopressors were independent risk factors for AKI [10-12]. Additionally, the use of medications such as vancomycin, nonsteroidal anti-inflammatory drugs (NSAIDs), diuretics, and renin-angiotensin system inhibitors (RASI) were also potentially avoidable risk factors [13]. Classifying patients according to COVID-19 disease catergories of mild, severe, and critically ill, the incidence rates of AKI were 0.1-2%, 3-3.2%, and 8.3-29%, respectively [14]. The varying rates are likely due to differences in study populations, with most including ill patients needing hospitalizations in the studies reporting higher AKI rates.

Data on AKI in COVID-19 from the Western Pacific region, including the Southeast Asian region, is lacking. In Southeast Asia, only one study from Singapore reported an incidence of 8.1% [4]. This current study of a prospectively maintained database reports the presentations and outcomes of AKI in COVID-19 from Brunei Darussalam, which had a low threshold for hospital admissions during the pandemic. The study's objectives were to; a) describe the incidence and outcomes of AKI, b) identify factors that may influence patient outcomes, and c) determine the pattern and types of AKI that may influence outcomes of AKI patients with COVID-19.

## Materials and methods

Study design

This was a retrospective study of patients admitted to the National Isolation Centre (NIC), Brunei Darussalam, the only designated hospital managing COVID-19 patients during the study period.

Setting

During the first and initial part of the second wave, all patients diagnosed with COVID-19 were admitted to the NIC for isolation and treatment, as per the national management protocol. Soon after the start of the second wave, Community Isolation Centers were opened to accommodate patients with milder diseases.

Study population

The study population included the entire population affected by SARS-CoV-2, hospitalized in the NIC during the second wave. All patients admitted to the NIC from 7th August to 30th September 2021 were considered for this study. This range of dates was chosen because all patients with COVID-19 in the country were required by law to be admitted to the NIC for observation and treatment during that time. 7th August was the date when the first local case of COVID-19 was reported in the second wave. 30th September was the date when the government decided to transfer low-risk cases to other healthcare facilities, where routine blood tests were not taken. The sampling methodology was total enumerative consecutive sampling, with a target of 1000 patients or when the study period ended.

Data

For the study, prospectively collected data used in the management of patients was anonymized and used for analyses. Data collected included demographic data (gender, age, race, and nationality), co-morbidities (including DM, HT, CKD, and vascular disease [VD]), vaccination status, symptoms manifestations collected using a template (including gastrointestinal symptoms; vomiting, and diarrhea), medications exposures, admission blood investigations, and outcomes. VD was defined as a history of cardiovascular, cerebrovascular, and peripheral vascular disease. Collected investigation parameters included complete blood count, renal profile (serum sodium, potassium, urea and creatinine), C-reactive protein (CRP), serum albumin, and cycle threshold (Ct) values of reverse-transcriptase polymerase chain reaction (RT-PCR). Medication exposure included nephrotoxic agents (RASI, NSAIDs, diuretics, and vancomycin) and antibiotics. Outcome events included AKI, intensive care admission, deaths, usage of KRT, and length of hospital stay. The outcome was defined as having occurred within 30 days of admission, and as a result of a direct long-term complication of infection. An adverse event was defined as death, intensive care admission, high dependency unit admission, and usage of KRT.

Inclusion and exclusion criteria

We included patients older than 18 years old admitted during the study period until 30 September 2021 (data freeze). All patients with pre-existing end stage kidney disease (ESKD) on KRT or with insufficient data for blood investigations were excluded from the study.

Outcomes

The Kidney Disease: Improving Global Outcomes (KDIGO) criteria for AKI were used for this study [15]. As urine output was not routinely collected for all patients, it was not included as a criterion for AKI. Patients identified with AKI were further sub-classified into Stage 1, Stage 2, or Stage 3. AKI was also categorized into temporal categories (AKI before admission and AKI during admission), and whether there was a history of CKD. The most recent serum creatinine values were considered as the baseline for patients with serum creatinine levels before admission. For patients without a baseline creatinine level before admission, the estimated creatinine was calculated using the Modification of Diet in Renal Disease (MDRD) study equation, assuming that baseline estimated glomerular filtration rate (eGFR) is 75ml/min per 1.73m2 [16].

Statistics

Statistical analyses were performed with R version 2.6.2 (R Foundation for Statistical Computing, Vienna, Austria). Continuous variables were expressed as means or median and standard deviation or interquartile range, whilst categorical variables were expressed as frequency and percentages. Means comparison was made using Independent T-test (normal distribution) or Mann-Whitney test (non-normal distribution) for two groups; and one-way ANOVA for multiple groups with Scheffe’s procedure for post hoc analysis. Levene’s test was used to assess variance in standard deviations of means. Pearson’s Chi-Square test or Fisher’s exact test determined the association between categorical or nominal variables. P-values < 0.05 were considered statistically significant. The crude odds ratio and adjusted odd ratio (with a 95% confidence interval) were calculated using simple logistic regression and multiple logistic regression, respectively. The variables for the multi-logistic model were identified through the stepwise elimination of insignificant variables through a forward and backward selection technique. Multicollinearity and interactions were assessed independently for variables included in the final model. Overall model fitness, to fulfill the assumption for binary logistic regression, was performed through Hosmer-Lemeshow goodness-of-fit test.

Ethical considerations

The study was approved by the Ministry of Health as part of a wider surveillance study during the acute phase of the pandemic. The study used prospectively collected data used in the management of patients that were anonymized for the study. All work was conducted in accordance with the guidelines of the Declaration of Helsinki. Ethical approval was given by the Medical Health and Research Ethic Committee, Ministry of Health (Reference MHREC/MOH/2023/08(1)).

## Results

A total of 1,000 patients were shortlisted through enumerative consecutive sampling and 70 were excluded because of ESKD (n = 8) and inadequate data (n = 62), leaving 930 patients in the study.

Demographics and characteristics

The mean age was 41.9 ± 14.4 years, with a male preponderance of 52.7% (n = 490). Among the comorbid conditions, DM, HT, VD, and CKD accounted for 11.7%, 29.1%, 5.6%, and 4.8%, respectively. Vaccination for COVID-19 was recorded at 5.2%. Gastrointestinal symptoms, particularly diarrhea and vomiting were reported by 16.2%. Exposure to nephrotoxic medications was recorded for 17.9%, either at the time of admission or during hospitalization. Table 1 shows the demographic and characteristics of the whole patient population and those with and without AKI.

**Table 1:** Comparisons between demographic factors and outcomes between AKI and non-AKI groups. AKI: acute kidney injury

		All patients n (%)	AKI -Yes n (%)	AKI-No n (%)	X2 Statistic	P-Value
All patients	Variables	930 (100%)	109 (11.7%)	821 (88.3%)		
Age (mean years ± standard deviation)		41.90 ± 14.37	53.26 ± 15.86	40.39 ± 14.97		<0.001
Gender	Male	490 (52.7%)	68 (13.9%)	422 (86.1%)	5.47	0.019
	Female	440 (47.3%)	46 (10.5%)	394 (89.5%)		
Race	Malay	771 (82.9%)	103 (13.4%)	668 (86.6%)	13.18	0.001
	Chinese	68 (7.3%)	5 (7.3%)	63 (92.7%)		
	Others	91 (9.8%)	1 (1.1%)	90 (98.9%)		
Nationality	Locals	775 (83.3%)	106 (13.7%)	669 (86.3%)	17.21	<0.001
	Foreigners	155 (16.7%)	3 (1.9%)	152 (98.1%)		
Diabetes mellitus	Yes	109 (11.7%)	46 (28.4%)	63 (8.2%)	52.71	<0.001
	No	821 (88.3%)	116 (8.2%)	705 (91.8%)		
Hypertension	Yes	271 (29.1%)	69 (25.5%)	202 (74.5%)	69.79	<0.001
	No	659 (70.9%)	40 (6.1%)	619 (93.9%)		
Vascular disease	Yes	52 (5.6%)	21 (40.4%)	31 (59.6%)	43.74	<0.001
	No	878 (94.4%)	88 (10.0%)	790 (90.0%)		
Presence of gastrointestinal symptoms (nausea and vomiting)	Yes	151 (16.2%)	29 (19.2%)	122 (80.8%)	9.76	0.002
	No	779 (83.8%)	80 (10.3%)	699 (89.7%)		
Nephrotoxins exposure	Yes	167 (18.0%)	61 (36.5%)	106 (63.5%)	121.06	<0.001
	No	763 (82.0%)	48 (6.3%)	715 (93.7%)		
Antibiotics exposure	Yes	292 (31.4%)	64 (21.9%)	228 (78.1%)	42.78	<0.001
	No	638 (68.6%)	45 (7.1%)	593 (92.9%)		
Vaccination	Full	48 (5.2%)	6 (12.5%)	42 (87.5%)	0.06	0.971
	Partial	142 (15.3%)	16 (11.3%)	126 (88.7%)		
	No	740 (79.6%)	87 (11.8%)	653 (88.2%)		
Death	Yes	42 (4.5%)	32 (76.2%)	10 (23.8%)	176.69	<0.001
	No	888 (95.5%)	77 (8.7%)	811 (91.3%)		
Intensive care admission	Yes	22 (2.4%)	18 (81.8%)	4 (18.2%)	100.18	<0.001
	No	908 (97.6%)	91 (10.0%)	817 (90.0%)		
Kidney replacement therapy	Yes	18 (1.9%)	17 (94.1%)	1 (5.9%)	105.66	<0.001
	No	912 (98.1%)	92 (10.1%)	820 (89.9%)		
Hospital stays	<10 days	409 (44.0%)	17 (4.2%)	392 (95.4%)	61.01	<0.001
	10-20 days	458 (49.3%)	70 (15.3%)	388 (84.7%)		
	> 20 days	63 (6.8%)	22 (34.9%)	41 (65.1%)		

Incidence of AKI

The incidence of AKI was 11.7% (n = 109); Stages 1, 2 and 3 occurred in 67.9%, 13.8% and 18.3%, respectively. AKI was present pre-admission in 75.2%, whilst 24.8% were first observed during hospitalization. Acute exacerbation of underlying CKD occurred in 26.6% of patients, whilst 73.4% were *de novo* AKI. Adverse events, deaths, and KRT usage occurred in 50.5%, 29.4%, and 15.6% of AKI patients, respectively.

Comparisons between AKI and non-AKI groups

Comparisons between the AKI and non-AKI groups revealed significant differences among the variables assessed. Higher proportion of AKI was encountered among the male gender, local nationality, Malay race, patients with DM, HT, VD, reporting GI symptoms, antibiotics usage, and nephrotoxin exposure (all p values <0.05). AKI patients were also significantly older (53.3 ± 15.9 vs 40.4 ± 14.9 years), but there was no difference in vaccination status. Table 1 shows the comparisons between demographic factors and outcomes between AKI and non-AKI groups.

Among the laboratory investigations, AKI patients had a significantly higher white cell count, serum potassium, urea, creatinine, and C-reactive protein levels; and lower platelet levels, sodium, and albumin levels. There was no significant relationship between hemoglobin levels and Ct values in RT-PCR. AKI patients were more likely to die, be admitted to the intensive care unit, require KRT and have a longer hospital stay. Table 2 shows the differences in blood parameters between AKI and non-AKI groups.

**Table 2:** Comparison of blood parameters between AKI and non-AKI groups. All figures presented as mean and standard deviations AKI: acute kidney injury

	AKI (mean ± standard deviation)	Non-AKI (mean ± standard deviation)	P value
Hemoglobin (gm/dL)	13.2 ± 2.3	13.8 ± 5.6	0.052
White cell count (x10^9^)	7.4 ± 3.3	6.3 ± 2.5	<0.001
Platelets (x10^9^)	221.1 ± 79.9	245.1 ± 88.6	0.007
Sodium (mmol/dL)	133.8 ± 4.6	135.2 ± 4.7	0.003
Potassium (mmol/dL)	4.2 ± 0.7	3.9 ± 0.4	<0.001
Urea (mmol/dL)	9.1 ± 8.3	3.4 ± 2.4	<0.001
Creatinine (mmol/dL)	186.6 ± 240.2	77.8 ± 49.5	<0.001
C-reactive protein (gm/dL)	6.5 ± 6.0	3.5 ± 4.6	<0.001
Albumin (gm/L)	32.9 ± 5.9	35.9 ± 5.1	<0.001
Cycle threshold (Ct) value	21.7 ± 6.8	21.2 ± 6.8	0.410

Univariate analysis identified age (OR 1.06), male gender (OR 1.63), local nationality (OR 8.03), DM (OR 4.44), HT (OR 5.29), VD (OR 6.08), GI symptoms (OR 2.08), antibiotic usage (OR 3.70) and nephrotoxin exposure (OR 8.57) as significant variables. Multi-logistic regression analysis showed that only age, male gender, GI symptoms, antibiotic usage, and nephrotoxin exposure remained significant in the final model with adjusted odds ratios of 1.04, 1.67, 1.61, 2.34, and 4.73, respectively. There were no significant interactions or multicollinearity between the final five variables in the model. Table 3 shows the univariate and multivariate analyses for risk factors.

**Table 3:** Crude odds ratio (COR) and adjusted odds ratio (AOR) for developing AKI AKI: acute kidney injury

Variable		Univariate analysis				Multivariate analysis			
		Crude Odds Ratio	95% Confidence interval	Z Stat	P value	Adjusted Odds Ratio	95% Confidence interval	Z Stat	P Value
Age (Year)		1.06	1.05, 1.08	8.18	<0.001	1.04	1.02, 1.06	4.56	<0.001
Gender	Male	1.63	1.08, 2.48	2.32	0.02	1.67	1.06, 2.66	2.20	0.028
	Female	1.00				1.00			
Nationality	Nationals	8.03	2.97, 32.92	3.52	<0.001				
	Expatriates	1.00							
Race	Malay	13.88	3.04, 245.91	2.60	0.009				
	Chinese	7.14	1.12, 138.62	1.78	0.075				
	Others	1.00							
Diabetes mellitus	Yes	4.44	2.88, 6.80	6.83	<0.001				
	No	1.00							
Hypertension	Yes	5.29	3.49, 8.11	7.76	<0.001				
	No	1.00							
Vascular disease	Yes	6.08	3.31, 10.99	5.94	<0.001				
	No	1.00							
Vaccination	Full	0.93	0.41, 2.50	-0.16	0.877				
	Partial	1.05	0.61, 1.91	0.17	0.868				
	No	1.00							
GastroiintestinaI symptoms	Yes	2.08	1.29, 3.08	3.07	0.002	1.61	0.91, 2.76	1.68	0.092
	No	1.00				1.00			
Nephrotoxins exposure	Yes	8.57	5.59, 13.23	9.80	<0.001	4.73	2.95, 7.61	6.45	<0.001
	No	1.00				1.00			
Antibiotics exposure	Yes	3.70	2.46, 5.61	6.24	<0.001	2.34	1.48, 3.71	3.63	<0.001
	No	1.00				1.00			

Sub-analysis of AKI revealed that AKI stages were incrementally associated with adverse outcomes; whilst timings (pre-admission and during admission) and presence of chronic kidney disease did not significantly cause adverse outcomes. Table 4 shows the types and patterns of AKI and their associations with adverse events.

**Table 4:** Types and patterns of AKI and associations with adverse events AKI: acute kidney injury

Variables		N (%)	Yes Adverse events	No Adverse events	X2 statistics	P value
AKI Stage	Stage 1	74 (67.9%)	24 (32.4%)	50 (67.6%)	32.40	<0.001
	Stage 2	15 (13.8%)	11 (73.3%)	4 (26.7%)		
	Stage 3	20 (18.3%)	20 (100%)	0 (0.0%)		
AKI- timing of diagnosis	Pre-admission	82 (75.2%)	38 (46.3%)	44 (53.7%)	2.24	0.134
	During admission	27 (24.8%)	17 (63.0%)	10 (37.0%)		
AKI	De novo	80 (73.4%)	36 (45.0%)	44 (55.0%)	3.58	0.058
	Acute on chronic	29 (26.6%)	19 (65.5%)	10 (34.5%)		

## Discussion

This study depicts the actual burden of AKI on COVID-19 patients in Brunei Darussalam, due to the admission and isolation policy at the time of the study. The incidence of AKI from this study was 11.7%, similar to the initial Chinese studies [1,5,14] with unselective admission policies. Our incidence is lower than those who have selective admission policies, where patients usually have more co-morbidities. Generally, findings from the east are in stark contrast to those from the rest of the world. A meta-analysis by Chan et al. reported lower incidence in Chinese provinces like Guangdong (1.74%), Hong Kong (3.72%), and Hubei (4.25%), but very high in American states like Michigan (44.73%), New York (33.07%) and Pennsylvania (49.33%) [17]. Similarly, other Western Pacific nations like Singapore (8.1%) and South Korea (4.0%) also reported low rates [4,18], compared to Middle Eastern countries (Iran 37.6%, Bahrain 47.6%) [19,20], Europe (Italy 22%, UK 29%, Turkey 54%, Portugal 55%) [21-24] and the Americas (USA 33-49%, Brazil 71%, Canada 54%) [6,7,25,26]. The wide discrepancies are likely related to dissimilarities in handling the pandemic through different screening and admission policies, healthcare disparities, hospital capacity, surveillance systems, social determinants, and disease epidemiology.

To date, only a handful of Asian countries have reported COVID-19-related AKI following the KDIGO classification for meaningful comparison with global data. A summary of studies involving Asian countries [4,18-20,27-29] is presented in Table 5. Our study’s AKI rate of 11.7% was higher than in some Asian countries, but this could be attributable to the higher prevalence of DM (16.7%) and HT (36.6%) in our patient population compared to the other Asian countries. The disease prevalence in our research population was similar to the overall national prevalence rates in 2020 (DM 13.5% and HT 28.0%) [16,30], which is expected given the non-selective nature of hospital admissions.

**Table 5:** Summary of Asian studies that list the incidence of AKI utilizing KDIGO criteria. AKI: acute kidney injury, KRT: kidney replacement therapy, DM: diabetes mellitus, HT: hypertension, CKD: chronic kidney disease, RASI: renin-angiotensin system inhibitors, NSAID: nonsteroidal anti-inflammatory drug, KDIGO: Kidney Disease: Improving Global Outcomes

Author	Origin	Study year	Number	Age (years)	Co-morbidities	Incidence	Outcomes	Significant Risk factors
Paek JH et al. [18]	South Korea	2020	704	57	18% DM, 32% HT	4.0% has AKI; Stage 1- 54%, Stage 2- 11%, Stage 3- 36%	8% need KRT, 28% mortality	Older age, males, DM, HT, chest x-ray findings
Zheng XZ et al. [27]	China	2020	555	52	10% DM, 28% HT, 2% CKD	5.3% has AKI; Stage 1- 38%, Stage 2- 19%, Stage 3- 43%	24% need KRT, 41% mortality	Proteinuria, hematuria and in-patient AKI
See YP et al. [4]	Singapore	2021	707	46	12% DM, 19% HT, 1% CKD	8.1% has AKI; Stage 1- 68%, Stage 2- 16%, Stage 3- 16%.		Older age, use of RASI, vancomycin, NSAID
Chan KW et al. [17]	Hong Kong	2021	644	38	6% DM, 14% HT, 9% CKD	3.7% with AKI; Stage 1- 59%, Stage 2- 14%, Stage 3- 27%	18.2% needed KRT, 13.6% mortality	Older age, smoking, DM, HT, CKD, use of RASI
N Naser et al. [19]	Bahrain	2021	353	56	Not stated	47.6% with AKI, Exact breakdown of the KDIGO AKI category is not stated, but mortality is correlated with staging	51.8% mortality	DM, HT
Rahimzadeh H et al. [20]	Iran	2021	516	58	32% DM, 41% HT, 4% CKD	37.6% with AKI; Stage 1- 62%, Stage 2- 18%, Stage 3- 20%	39.7% mortality	Male, CKD, HT, disease severity
Sindhu C et al. [29]	India	2022	2650	63 (AKI patients only)	72% DM, 67% HT, 23% CKD (AKI patients only)	7.2% with AKI; Stage 1- 71%, Stage 2- 15%, Stage 3- 14%	75% require KRT, 22.1% mortality	Older age, stage 3 AKI, mechanical ventilation
Tan J et al. (Current study)	Brunei	2022	930	42	17% DM, 37% HT, 5% CKD	11.7% has AKI; Stage 1- 68%, Stage 2- 14%, Stage 3- 18%	15.6% need KRT, 29.4% mortality	Older age, male, nephrotoxin usage, antibiotic usage, GI symptoms

Multivariate analysis identified five factors independently associated with AKI: advanced age, male gender, exposure to nephrotoxins, antibiotic exposure, and presence of GI symptoms (nausea and vomiting). Older patients are more likely to have advanced chronic changes in the kidneys (i.e., glomerulosclerosis, tubular atrophy, interstitial fibrosis, and arteriosclerosis of small vessels) [31], which can increase the propensity for severe kidney disease and related deaths [14]. The association between AKI and male gender is well described [32], with possible explanations being the protective effect of estrogen through the reduction of post-ischemic glomerular endothelial hyperpermeability and suppression of enhanced renal sympathetic nerve activity during renal ischemia [33,34]. Gl symptoms, besides causing intravascular depletion and compromised hemodynamic circulation, can also cause multiple electrolyte abnormalities (hypokalemia and hyponatremia), which compound early renal insufficiency leading to rapid progression of renal impairment [35]. Inadvertent exposure to nephrotoxins is likely to increase predisposition to AKI [36], increase oxidative stress in proximal renal tubular cells (vancomycin) [37], inhibition of prostaglandin leading to afferent arteriole vasodilatation (NSAIDs) [38] and dysregulation of renal hemodynamics through an aberration of salt and water retention (diuretics) [39]. Antibiotics use in this study was highly correlated with high admission CRP and white cell count (WCC), indicating that AKI has occurred due to COVID-19 or co-infection rather than the nephrotoxic effects of antibiotics. Bacterial co-infection with SARS-CoV-2 is associated with less favorable outcomes and prognosis.

Co-morbid conditions like DM, HT, and VD were also identified as risk factors. The univariate analysis confirmed the significant associations between individual co-morbidity with AKI. Still, the co-existence of these diseases in clustered groups probably canceled out their individual effects in the multivariate analysis. Underlying renal endothelial dysfunction, metabolic consequences of glucose dysregulation, and intraglomerular pressure changes in patients with these co-morbidities lower the threshold for AKI and increase vulnerability to adverse outcomes [40]. Foreigners (mainly nationals from the Philippines and Indonesia) in the country tended to have less AKI, but this was likely to be related to them being employable, younger workers, and having fewer co-morbidities. Patients of Malay ethnicity also had higher AKI rates, likely due to the higher prevalence of DM compared to Chinese and other races (19% vs 13% and 4%).

Among patients with AKI, stages 1, 2, and 3 accounted for 68%, 14%, and 18%, respectively. These are consistent with literature references amongst countries with lower AKI rates (Table 5). The relatively high incidence of Stage 1 disease, the low death rate (29.4%), and the low KRT usage rate (15.6%) in our AKI population could be explained by a high proportion of asymptomatic patients in the study population, which resulted in many mild cases of AKI being incidentally diagnosed on routine blood tests. There was no temporal relationship between early (pre-admission) and late (during admission) AKI and outcomes, which indicates that the reversibility of disease was limited once the pathological processes commenced, placing greater emphasis on the need for preventative measures in the community.

Few studies have reported the temporal relationship between the onset of SARS-CoV-2 infection and the development of AKI. Bendall et al. in 2022 [41] reported that community-acquired AKI has poorer outcomes, longer hospital stays and greater propensity for CKD compared to hospital-acquired AKI in non-COVID-19 patients. Another study on COVID-19 patients however showed that mortality is increased in hospital-acquired AKI compared to community-acquired AKI [42]. Establishing a time-related association is important because it can help to determine whether AKI is associated with a high viral load of SARS-CoV-2, especially at the start of the infection, or from other systemic manifestations, secondary infections, or iatrogenic hospital factors. Hirsch et al. reported that about one-third of patients presented with AKI or developed AKI within 24 hours of presentation [6], whilst another study reported a delay in the presentation of AKI (median of 15 days) [43]. Our study showed that most patients presented with early AKI, but there was no relationship between viral load and occurrence and onset of AKI, indicating that high viral replication at the time of admission did not have an impact on AKI.

Several limitations need to be considered when interpreting our findings. The study relied on secondary data entered by the clinical management team into a database to aid the management of COVID-19. Many patients (n = 62) were excluded from the final shortlist owing to incomplete data from early discharge or disposition to other quarantine facilities. Including these patients, presumably asymptomatic with no kidney injury, may have reduced the incidence of AKI in this study. Data for urine output and urinalysis was not routinely available as it was not part of the management protocol; hence some milder cases of AKI might not have been listed. Data that indicate severity of AKI like vasopressor usage, ventilatory requirements and disseminated intravascular coagulation profile should have been included at the outset of data collection as they may influence outcomes. We also could not assess renal function recovery as many patients did not have repeated kidney function tests or were transferred to other low-care quarantine facilities for further management or rehabilitation. The study was conducted during the 'containment' phase, whereby all COVID-19 patients were unselectively admitted; pandemic management has subsequently evolved to 'mitigation' and 'endemic' phases, where only higher-risked patients were admitted. Given the dynamic stages of pandemic management, the findings from this study might not be generalizable to all hospitalized patients with COVID-19 in the country. In addition, due to the study's observational nature, we could only establish associations rather than cause-effect relationships.

Despite this, the study highlights several important points in managing AKI in COVID-19 patients. The independent factors identified suggest that there were reversible and avoidable elements that could be addressed and rectified. With attention to specific details, we believe that the incidence and severity of AKI can be averted or diminished. Our findings, although based on COVID-19 patients affected by the Delta strain, can be used for future viral outbreaks or pandemics that share similar manifestations.

## Conclusions

This study provides a unique perspective on AKI in a population that encapsulates the entire disease spectrum of COVID-19 due to the country's prevailing isolation and admission policy. The incidence of AKI among patients with COVID-19 in Brunei Darussalam is comparable to other Asian countries but is much lower than in the West and the Middle East, with significant non-modifiable (advanced age and male gender) and modifiable (presence of GI symptoms, antibiotics usage, and nephrotoxin exposure) variables. 
